# Fabrication of Superconducting Nanowires Using the Template Method

**DOI:** 10.3390/nano11081970

**Published:** 2021-07-31

**Authors:** Michael Rudolf Koblischka, Anjela Koblischka-Veneva

**Affiliations:** 1 Experimental Physics, Saarland University, P.O. Box 151150, D-66041 Saarbrücken, Germany; anjela@shibaura-it.ac.jp; 2 Shibaura Research Laboratories, Shibaura Institute of Technology, 1-3-5 Toyosu, Koto-ku, Tokyo 135-8548, Japan

**Keywords:** superconductors, nanowires, templating, alumina (AAO) templates

## Abstract

The fabrication and characterization of superconducting nanowires fabricated by the anodic aluminium oxide (AAO) template technique has been reviewed. This templating method was applied to conventional metallic superconductors, as well as to several high-temperature superconductors (HTSc). For filling the templates with superconducting material, several different techniques have been applied in the literature, including electrodeposition, sol-gel techniques, sputtering, and melting. Here, we discuss the various superconducting materials employed and the results obtained. The arising problems in the fabrication process and the difficulties concerning the separation of the nanowires from the templates are pointed out in detail. Furthermore, we compare HTSc nanowires prepared by AAO templating and electrospinning with each other, and give an outlook to further research directions.

## 1. Introduction

Superconducting nanowires are interesting mesoscopic 1-dimensional (1D) objects for many reasons. Nanostructuring superconducting materials may show effects which are not known from the respective bulk materials: As examples, one may mention Pb films, where the transition temperature was found to show an oscillating behavior, depending on the thickness of the superconducting film [1] and Al nanowires, exhibiting size-dependent breakdowns of superconductivity [2]. Other superconducting materials, such as Ga, In, or Tl, may exhibit increased values of the superconducting transition temperature, $T_{c}$, when being prepared as quasi 0-dimensional (0D) nanoparticles of ultra-fine nanogranular materials [3,4,5,6,7,8].

From a fundamental point of view, superconductivity is characterized by two critical lengths [9,10], the London penetration depth, $\lambda_{L}$, and the coherence length, ξ, so one may prepare nanowires with at least one dimension below one of these characteristic lengths. A wire is classified as 1D if its diameter *d* is smaller than the superconducting coherence length ξ. As result, the superconducting properties may be different from what is obtained from bulk samples of the same composition, and quantum fluctuations [11,12] may dominate the superconducting properties [13,14].

From an application point of view, the interest in superconducting nanowires is pushed by the continuing drive for miniaturization in the electronics industry demanding the reduction of heat dissipation. This may require the use of superconducting interconnects between the semiconducting circuits [15]. Furthermore, the use of superconducting nanowires as functional elements in sensors, e.g., single photon detectors, is another big field of interest, especially in the field of quantum photonics [16,17,18,19]. And, finally, a new challenging and promising application for semiconducting or superconducting nanowires may be the hosting of qubits for quantum computing with improved stability [20].

In conventional superconductors, such as Al, Pb, Sn, Nb, NbN, MoGe, and others, in thin film or nanowire form, the coherence length is ξ∼ 5–100 nm, which is typically 10–1000 times the Fermi wavelength. In such wires, the wave function of the Cooper pairs only depends on the position along the wire, while it is independent of the position within the wire cross section. A nanowire is classified as quasi-one-dimensional (quasi-1D) if the diameter is of the order of $d<\pi \sqrt{2}\xi$. This condition ensures that vortices, having a core diameter of 2ξ, are not energetically stable in the wire. This means that vortices cannot penetrate into the nanowire, and the nanowire remains in the Meissner state even in large magnetic fields. Therefore, the superconducting order parameter is approximately constant within the cross section of the nanowire. Since ξ diverges at the critical temperature, $T_{c}$, it is not too complicated to fabricate a nanowire which is quasi-1D near $T_{c}$. Thus, the fabrication and characterization of such nanowires, mostly obtained by lithographical techniques from thin films on substrates, were discussed extensively in the books by Bezryadin [13] and Altomare [14].

In the case of high-temperature superconductors (HTSc), the superconducting parameters $\lambda_{L}$ and ξ are fundamentally different, as ξ is in the lower nanometer range (typical dimensions are $\xi_{ab}\left(0\right)∼$1.3 nm) and $\lambda_{L}\left(0\right)$ is very large (∼130 nm), owing to the fact that the Ginzburg-Landau parameter $\kappa_{\mathrm{GL}}$ is very large for the HTSc materials [21]. Thus, the fabrication of 1D-nanowires of HTSc materials is a large challenge, bringing the commonly used lithography techniques to their limits. The HTSc materials prepared in nanowire form comprise mainly YBa${}_{2}$Cu${}_{3}$O${}_{7-\delta }$ (YBCO) and Bi${}_{2}$Sr${}_{2}$CaCu${}_{2}$O${}_{8+\delta }$ (Bi-2212), both being the most studied cuprate HTSc in the literature. Furthermore, the influence of the chosen substrate on the superconducting properties of the resulting nanowires may play an important role, so substrate-free, freestanding nanowires are desired for many characterization measurements.

For this reason, approaches of nanotechnology became very interesting to the community. Several types of metallic and oxidic materials were prepared in nanowire form using track-etched polymer membranes and the alumina template (anodic aluminium oxide (AAO)) approach. This research was reviewed already in References [22,23,24,25,26,27].

Starting from the year 1997, magnetic and superconducting materials were fabricated using the template approach [28,29,30,31]. Both classes of materials share the presence of critical lengths, such as the domain wall width and the superparamagnetic limit for magnetic materials, and $\lambda_{L}$ and ξ for superconducting materials. Due to the actual research on patterned media for magnetic storage, magnetic materials (metallic ones, as well as ceramic-based ones) were fabricated as nanowires within the templates but also as films on top of the templates to serve as simple and cheap means to fabricate patterned media. This provided the base for research concerning superconducting nanowires. Here, it is important to mention that the templating approach is not limited to the aforementioned track-etched polymer membranes (soft templates) and the AAO templates (hard templates) but other types of templates, such as carbon nanotubes, block copolymers, biologic nanostructures, and others, were also used in the literature to grow magnetic and superconducting nanowires.

The fabrication processes of AAO templates and the self-organization of the pores were already extensively reviewed in the literature [32,33,34,35], and the fabrication of AAO-templated magnetic nanowires by electrodeposition was recently reviewed by Piraux [36]. Thus, in the present review, we focus on the fabrication processes of superconducting nanowires using mainly the AAO template approach, discuss the various problems appearing in the preparation and separation of the nanowires, and give an outlook to further research.

## 2. Basic Ideas

**Figure 1:**
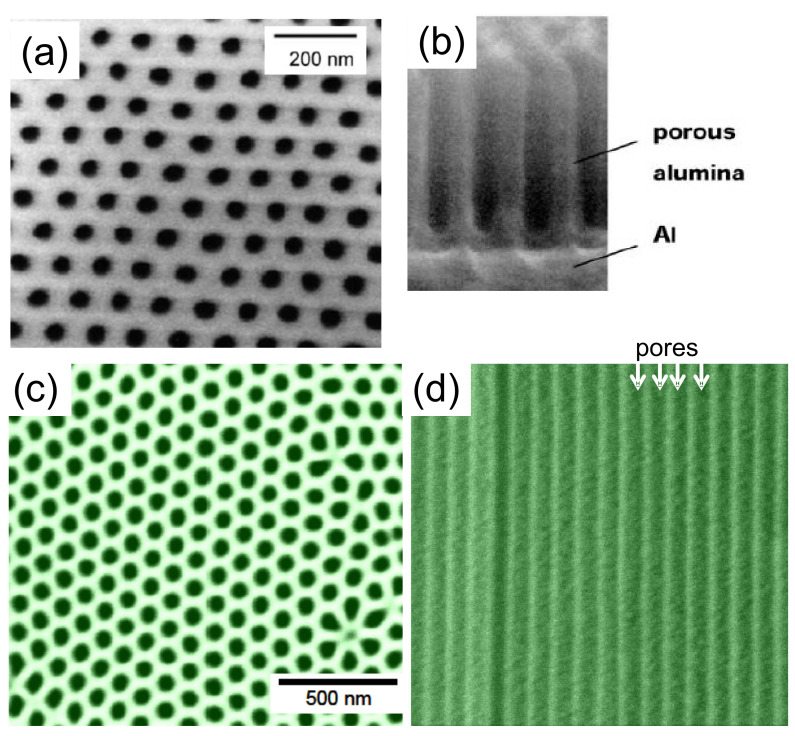
SEM images of anodic alumina oxide (AAO) templates. (**a**) Top-view of a self-fabricated AAO template and view of the cross section (**b**), illustrating the porous alumina layer and the Al${}_{2}$O${}_{3}$/Al underlayer (Reprinted with permission from Ref. [32]. Copyright 2003 Springer Nature). For comparison, a commercial AAO template (Whatman anodisc™, unframed, nominal pore diameter 100 nm [37]) is presented as top-view (**c**) and cross section (**d**).

**Figure 2:**
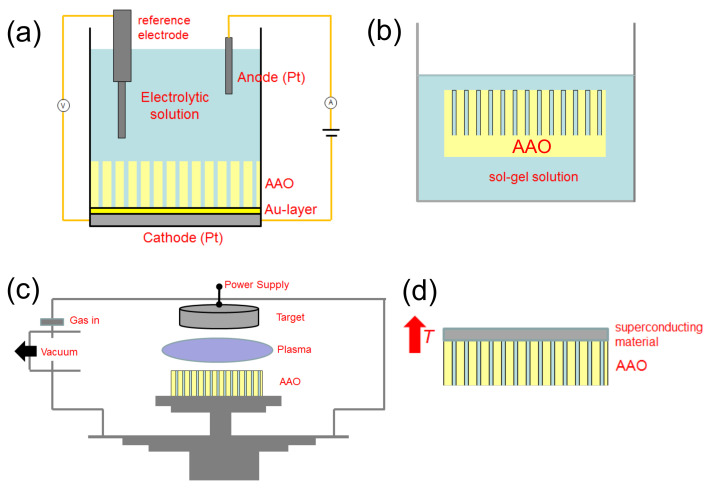
Schemes for filling the porous template material. (**a**) electrodeposition, (**b**) sol-gel solution, (**c**) sputtering, and (**d**) melting. AAO indicates the anodic alumina oxide template as an example. In case (**b**), the alumina oxide at the bottom of the template is useful, whereas it must be removed in (**a**).

The basic idea of the fabrication of superconducting nanowires via the AAO template approach is to fill the template with superconducting material. Figure 1 presents SEM images of empty AAO templates. Figure 1a (top view) and Figure 1b (cross section) stem from a self-prepared template with the Al-layer at the bottom, and Figure 1c (top view) and Figure 1d (cross section) belong to a commercial AAO template (Whatman anodisc™ [37]). Now, the pores may be filled up using various possible techniques, such as electrodeposition, sol-gel processes, sputtering, and melting, were reported in the literature [38,39,40,41]. With some apparative arrangements, classic deposition techniques, such as evaporation or sputtering, may also be employed to fill the templates [42]. This is illustrated schematically in Figure 2a–d. As electrodeposition [43] (a) is a quite flexible technique, the fabrication of multi-layered systems, such as Nb/Cu nanowires is also possible. The combination of electrodeposition and AAO templates is, thus, a very useful one, but is mainly done on conventional superconductors, even though YBCO can also be fabricated by electrodeposition [44,45]. The filling of the AAO pores using the sol-gel approach (b) is very interesting especially for ceramic materials, such as the HTSc. Figure 2c shows the arrangement for sputtering as an example. In this situation, the pores may not be completely filled with material, and there will be in all cases a remaining top layer of the sputtered material, which must be properly removed before preparing any electric contacts. Figure 2d illustrates the melting approach. Here, the material to be filled into the templates is put on top, and the temperature is increased above the melting point. The then molten material may enter into the pores of the template. This approach has been used in the literature to fabricate YBCO nanowires.

Besides the filling of the nanopores with a superconducting material, the hexagonal lattice of the pores in the AAO templates is very similar to the heagonally ordered vortex lattic formed in type-II superconductors in high magnetic fields. Thus, there is a possibility to study matching effects of the vortex lattice in a superconducting film evaporated on top of the AAO template with the lattice of the AAO pores. In this case, there is no need to remove anything from the templates similar to the patterned media in magnetism. These matching effects can even be enhanced when filling the AAO templates with a magnetic material, such as Ni, Fe, or Co, and then covering this template with a superconducting film. Therefore, this second approach has also attracted researchers to these effects. Of course, such experiments were carried out using conventional metallic type-II superconductors, such as Nb, NbN, or MoGe [46,47,48,49,50,51], as AAO is not a reasonable template for HTSc thin films. These experiments will be discussed in Section 3.4 below.

## 3. Realizations

### 3.1. Conventional Superconductors

In this section, we will have a look at the various conventional superconductors prepared in nanowire form using AAO templates. Dubois et al. [29] and Yi and Schwarzacher [31] were the first to report on superconducting Pb nanowires applying the templating technique; both groups using track-etched polycarbonate membranes, the electrodeposition technique and Pb as superconducting material.

**Figure 3:**
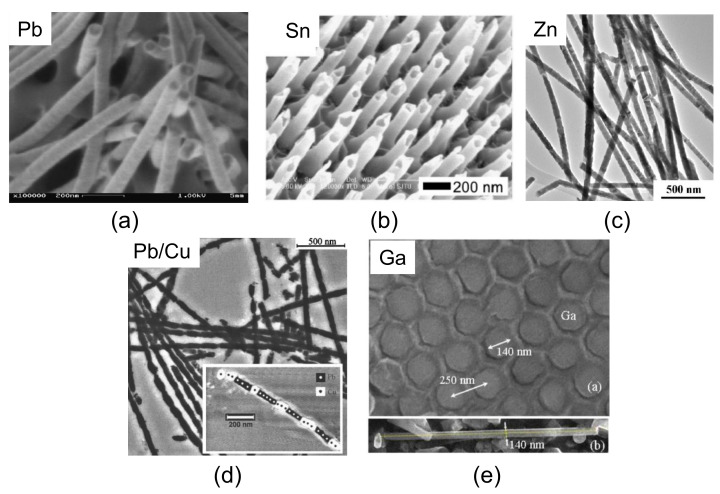
SEM micrographs of various superconducting nanowires of conventional metallic superconductors (**a**) Pb, (**b**) Sn, (**c**) Zn, (**d**) Pb/Cu, and (**e**) Ga. The insets to (**d**,**e**) give details of an individual nanowire; (**a**,**b**) are reprinted with permission from Elsevier [8,52,53,54], (**c**) is Reprinted (Adapted) with permission from Ref. [38]. Copyright 2005 American Chemical Society, (**d**) is Reprinted with permission from Ref. [54] Copyright 2005 AIP Publishing, and (**e**) is reprinted with permission from Ref. [8]. Copyright 2017 Springer Nature.

Figure 3 presents SEM images of superconducting nanowires of metallic type, such as Pb (a), Sn (b), Zn (c), Pb/Cu (d), and Ga (e) [8,38,52,53,54]. For all these elemental superconductors, the wire diameter is clearly below the critical dimensions; thus, electric transport measurements may reveal the 1D character. Note that most of these elemental superconductors are also type-I superconducting materials. Consequently, already, the first experiment on superconducting nanowires [29] showed a large enhancement of the critical field of the Pb nanowire arrays fabricated. Further work focused then on effects of the 1D nature of the nanowires, manifested by a non-zero resistance, which represents different stages within the superconducting state. A possible explanation of this behavior is the formation of phase-slip centers when the current of a magnetic field destroys superconductivity. Thus, electric transport measurements (magneto-resistance, *I*/*V*-characteristics are very important to analyze these properties of the nanowire arrays or of extracted, individual nanowires [52,55,56]. Therefore, in Ref. [57], the group from Louvain described a method based on nanolithography techniques to prepare proper electric contacts to the nanowire arrays, which is useful for all electric transport measurements on the nanowire arrays.

Zhang and Dai [58] fabricated 45 nm-diameter Pb nanowires by electrodopsition in AAO templates. They found the Pb nanowires to exhibit fcc structure and a structurally uniform behavior. The anisotropic magnetic properties (magnetic fields applied parallel and perpendicular to the AAO template) were measured by SQUID magnetometry, revealing the behavior of Pb as a type-II superconductor with a $T_{c}$ just below 7 K. Furthermore, they found flux entry and exit to the sample being inhibited above the lower critical field, $H_{c1}$, when increasing/descreasing the applied magnetic field.

Multilayered superconductors (mainly Nb/Cu thin films) were intensively investigated in the literature [59,60], so it was very straightforward to prepare electrodeposited, multilayered nanowires using the AAO template approach. de Menten de Horne [61] applied a single bath technique to fabricate Pb/Cu multilayered nanowires and achieved a relatively good control of the geometrical parameters with Cu layers as thick as 10 nm. A very specific pattern of the magnetoresistance was obtained in low magnetic fields, which is likely to be caused by the proximity effects, demonstrating the interesting physics behind such multilayered nanowires.

Li et al. [53] have applied DC magnetron sputtering to prepare Sn nanowire arrays in AAO templates and measured magnetic moments as function of temperature and field at temperatures down to 2 K. Furthermore, electron microscopy was employed to study the resulting microstructures in detail. Two different types of morphologies were obtained due to control of the substrate temperature during the sputtering process. The Sn film deposited onto the AAO template at room temperature was found to exhibit a wet property and produced cross-linked Sn nanotube arrays. In contrast to this behavior, isolated Sn nanotube arrays were obtained with an increased substrate temperature. This finding demonstrates that sputtering can also be employed to fabricate superconducting nanowire arrays, and, eventually, multilayered systems, as well, such as Sn-Pt.

Even Ru nanowires were fabricated by Wang et al. [62] using the template approach, employing commercial, track-etched polycarbonate membranes with 30 and 50 nm pores. The Ru nanowires were found to be polycrystalline, consisting of ultrasmall grains with only 2 nm diameter. The electric transport measurements performed showed that the nanowires were metallic, but no superconductivity was found at temperatures down to 0.3 K (the expected $T_{c}$ value of Ru bulk material is 0.51 K), which may due to the very small grain size.

Samples of nanostructured β-Ga wires were successfully prepared by a novel method of metallic-flux nanonucleation in AAO templates by Moura et al. [8], allowing the determination of several superconducting parameters via magnetic measurements. The authors could well describe the Ga nanowires as a weak-coupling type-II-like superconductor with a Ginzburg-Landau parameter $\kappa_{GL}=$ 1.18, favorized by the nanoscopic scale of the Ga nanowires. This result, including the measured relatively high $T_{c}$ of 6.2 K, is in stark contrast to pure bulk Ga, which is a type-I superconductor with a $T_{c}$ of ∼1.08 K [63,64]. Thus, it is worth it to investigate the $T_{c}$ increase as a function of nanowire diameter in future works.

Another interesting experiment concerning superconductivity and AAO templates was carried out by Haruyama et al. [65], who synthesized multi-walled carbon nanotubes (MWNTs) in AAO templates using chemical vapor deposition (CVD). The rigid AAO template served as holder to cut off the ends of the MWNTs by ultrasound, enabling to evaporate gold/Nb electrodes to the open ends of the MWNTs. The appearance of proximity-induced superconductivity and supercurrents in the MWNTs evidenced the high quality of the contacts prepared.

To summarize this section, it is obvious that nanowires of elemental superconductors are very interesting objects providing new physics of the superconducting state, so the research in this direction is ongoing and will include even more materials, such as In, Sn using the metallic-flux nanonucleation technique, or some types of metallic alloys.

### 3.2. High-Temperature Superconductors (HTSc)

**Figure 4:**
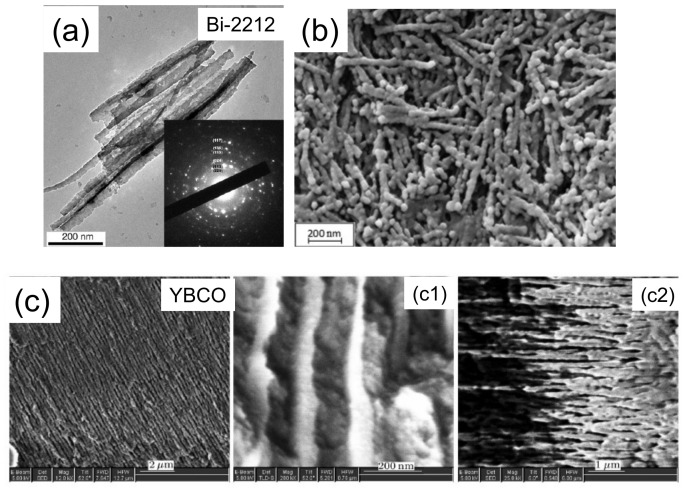
SEM images of (**a**) Bi-2212 nanowires [75] (Reprinted with permission from Ref. [75]. Copyright 2005 Elsevier), (**b**) YBCO nanowires produced with sol-gel approach [70] (Reprinted with permission from Ref. [70]. Copyright 2015 Elsevier), and (**c**) AAO template (low magnification) with YBCO nanowires prepared from molten YBCO powder, (**c1**) YBCO nanowires with high magnification and (**c2**) gives details about the distribution of the nanowires within the template [40] (Reprinted with permission from Ref. [40]. Copyright 2005 Chin. Phys. Lett.).

**Figure 5:**
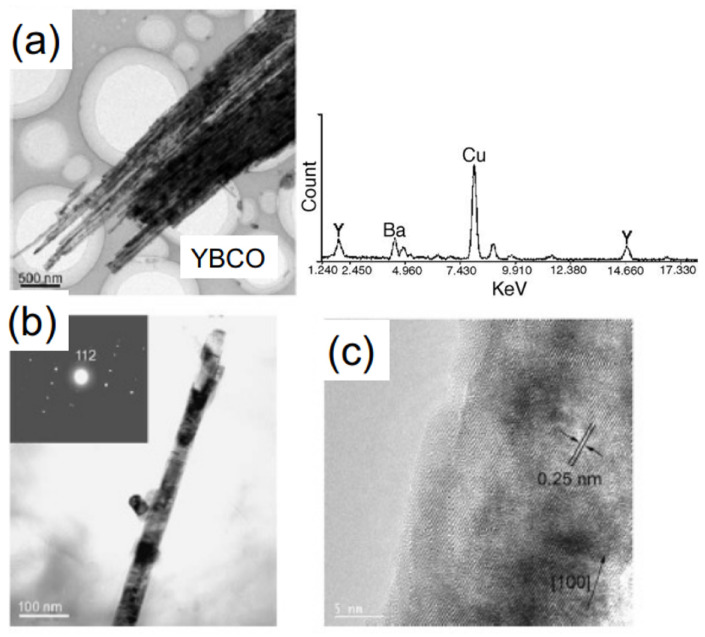
YBCO nanowires prepared by AAO-templating, released from the AAO template (**a**). The EDX spectrum on the right side did not reveal Al; (**b**) gives a TEM image of a representative YBCO nanowire with a diameter of ∼45 nm and a length up to the thickness of the AAO template. The inset shows the corresponding electron diffraction pattern, demonstrating the single-crystalline nature of the YBCO nanowires; (**c**) shows the lattice structure of an individual YBCO nanowire by HRTEM. Reprinted with permission from Ref. [39]. Copyright 2004 Elsevier.

To prepare nanowires of HTSc materials, the AAO template approach was also used several times in the literature to grow YBa${}_{2}$Cu${}_{3}$O${}_{7}$ (YBCO), NdBa${}_{2}$Cu${}_{3}$O${}_{7}$ (NdBCO), and Bi${}_{2}$Sr${}_{2}$CaCu${}_{2}$O${}_{8}$ (Bi-2212) nanowires. Several examples are presented in Figure 4 and Figure 5. Two approaches were employed, the sol-gel technique and the melting one. The sol-gel route is very useful to prepare ceramic materials in a controlled manner, and many experiments are described in the literature to grow HTSc superconducting materials in this way [39,66,67,68,69,70]. The melting approach uses pre-prepared HTSc powder on top of the template, which is then heat-treated above the melting temperature. This approach offers the possibility to reduce the melting temperature by using superconducting nanopowders.

However, there are two main problems arising: (i) the necessary etching away of the AAO template material after the fabrication process is difficult without affecting the HTSc material, and (ii) the Al${}_{2}$O${}_{3}$ material itself may have an effect on the resulting superconducting properties of the nanowires to to diffusion of Al into the HTSc cell. The latter point was found already during the first preparation of YBCO single crystals using Al${}_{2}$O${}_{3}$ crucibles [71], when the first large YBCO crystals obtained showed only a $T_{c}$ of ∼65 K. A consequence of this is that the contact time of a molten HTSc with the AAO template should be as short as possible, which excludes the application of a temperature program for the growth of single crystalline material. Furthermore, the AAO templates are hardly stable in such a process. Therefore, single crystal-type nanowires cannot be fabricated using the melting approach, and the resulting nanowires are polycrystalline with many small HTSc grains. The former point is even more problematic: It is practically impossible to find an etching solution which does not affect the HTSc material itself. All the etchants employed to extract the superconducting metallic nanowires or the magnetic nanowires described in the literature will also attack the HTSc material. So, the best solution for investigation of the superconducting properties is to study the nanowire arrays in the entire filled template without attempting to extract the nanowires.

A well-suited method to remove and cut superconducting nanowires is the focused ion-beam milling (FIB) technique [72]. However, it the case of the templated superconductors, it is only possible to cut some sections, but it is unsuitable to remove the nanowires from the templates. In case of HTSc superconductors, the alumina is also not a well-suited substrate material as Al may substitute for Cu in the Cu-O-planes, as already mentioned before.

Xu et al. [39] applied the sol-gel route to fill the AAO pores. Their main achievement is the finding that, at certain temperatures of the gels, single-crystalline YBCO nanowires could be obtained, which is very interesting for electric and magnetic measurements. The sol with dispersed blue colloidal particles was kept at a temperature of 70 ${}^{\circ }$C, and the AAO template was dipped into the hot sol. As consequence, a well crystallized YBCO phase was obtained at about 700 ${}^{\circ }$C, which is the lowest temperature reported in the literature compared to the temperatures applied in the ceramic method [73] and other sol–gel processes for YBCO [39,70,74]. Thus, this reduction of the fabrication temperature is a very important step in order to fabricate YBCO nanowires with the AAO templates. However, in their paper, these authors did not show any kind of measurement of the superconducting properties of the nanowires. Dadras and Aawani [70] also dipped AAO templates into an YBCO sol, as well as applied the melting approach with pre-prepared YBCO powder. For both types of samples, they could successfully remove the nanowires from the templates by NaOH etching.

Zhang et al. [74] were employing the sol-gel route and discussed the growth process of YBCO within the AAO templates in detail. In their optimized sol–gel process based on the Pechini method, molecular level mixing was carried out in the form of Y–Ba–Cu–EDTA (ethylenediamine tetraacetic acid) complex and the network subsequently formed by esterification of ethylene glycol and the metal–EDTA complex.

Lai et al. [75] successfully used the sol-gel technique and AAO templates to prepare Bi-2212 nanowires (Figure 4). The magnetically determined (zero-field cooling (ZFC), 10 Oe) transition temperature was 84 K, which is close to the bulk value. Furthermore, the authors presented magnetization measurements at various temperatures (2, 5, 20, and 50 K) in the field range ±70 kOe, which show all the features of a polycrystalline material. The magnetic signals at more elevated temperatures were too small to be measured with their setup.

Li et al. [40] were the first to apply the filling of the AAO templated by melting liquid in order to produce YBCO nanowire arrays. These authors attempted to separate the YBCO nanowires from the templates but failed to do so. Thus, they could only characterize the superconducting properties of the entire nanowire arrays using an AC susceptibility technique and found the $T_{c}∼$91 K, which directly corresponds to the bulk value. However, the superconducting transition width is very broad and incomplete down to 80 K. The measured $T_{c}$ and the x-ray data confirmed that YBCO is properly formed in this melting approach.

To summarize this section, we can state that is well possible to fabricate YBCO and Bi-2212 nanowire (arrays) using the AAO template approach. All authors characterized the microstructure of their samples using electron microscopy (SEM, EDX, TEM) and X-ray diffraction (Figure 5), but more intensive analysis was not done due to the problems of extracting the HTSc nanowires from the templates. Thus, electric transport measurements or magnetization data were also hardly collected, and if, then only for full nanowire arrays. Thus, the possible interesting physics of such HTSc nanowires has not yet been addressed properly in the literature.

### 3.3. Filling Commercial AAO Templates with High-$T_{c}$ Superconducting Materials

**Figure 6:**
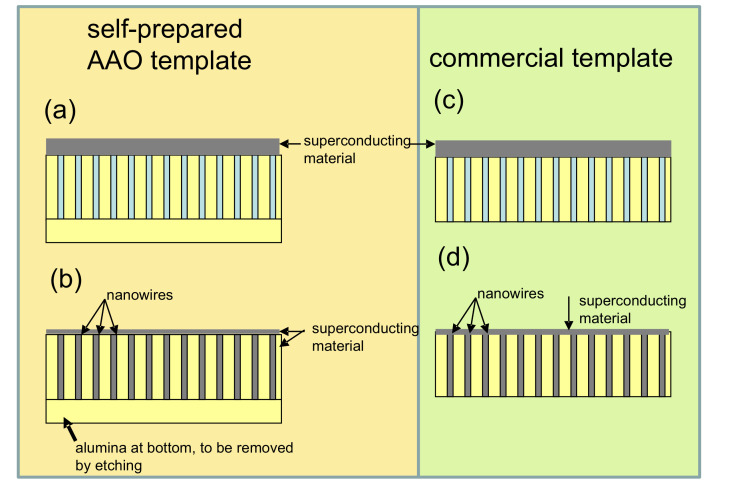
(**a**–**d**) Schematic drawing of the steps in the template technique; (**a**,**b**) present the situation for a self-prepared AAO template. The superconducting material (grey) is placed on top of the template (empty pores are indicated in light blue), and the pores are filled (indicated in dark gray) with the molten material. Images (**c**,**d**) give the situation for a commercial template. Note that, in both (**b**,**d**), the remaining superconducting material covers the top of the template. In (**b**), the untreated alumina bottom must also be removed after the processing, if electrical contacts to the nanowires are required.

The use of commercially available AAO templates (Whatman anodisc™ [37]) for the fabrication of HTSc nanowires is quite attractive, offering a possible nanowire diameter as small as 20 nm, which would bring the nanowire diameter close to the critical dimensions, especially at elevated temperatures close to $T_{c}$. Figure 6 gives a schematic view of the differences between the commercial AAO templates and the self-fabricated ones. As the anodisc templates are open on both sides according to their main use as nanofilters, there is no need to remove the Al/Al${}_{2}$O${}_{3}$ layer at the bottom. Using the melting approach, the missing bottom layer is not a problem as the template can be placed on a suitable underlayer. The templates have a thickness of 50 μm and an overall diameter of 27 mm, and the nanopores run through the entire thickness of the template. Thus, we attempted to grow YBCO and NdBa${}_{2}$Cu${}_{3}$O${}_{y}$ (NdBCO) nanowires using the anodisc templates, and the results were published in Refs. [41,76,77]. Pre-reacted YBCO and NdBCO powders were employed, ground to an average particle size of about 1 μm by ball-milling. The preparation of such powders was described by Hari Babu et al. [78]. An Al${}_{2}$O${}_{3}$ plate was placed on top of the template/powder arrangement, and at the bottom, a plate of the “green phase” Y${}_{2}$BaCuO${}_{5}$ (Y-211) was used to have a reaction-free underlayer. The entire heat treatment took place in a standard laboratory box-type furnace. The heat treatment was chosen similar to the single crystal growth process [79], but with a longer holding time of the maximum temperature (1050 ${}^{\circ }$C for YBCO, 1100 ${}^{\circ }$C for NdBCO) in order to ensure the complete melting of the powder. For the following electric and magnetic measurements, an oxygenation step was applied (450 ${}^{\circ }$C, 12 h, flowing O${}_{2}$). The resulting HTSc-filled AAO templates were found to be very brittle and break easily into several pieces. Thus, for the further handling steps, the pieces of the HTSc-filled templates were glued on Macor™ plates with GE varnish, enabling even mechanical grinding and polishing of the HTSc-filled templates.

**Figure 7:**
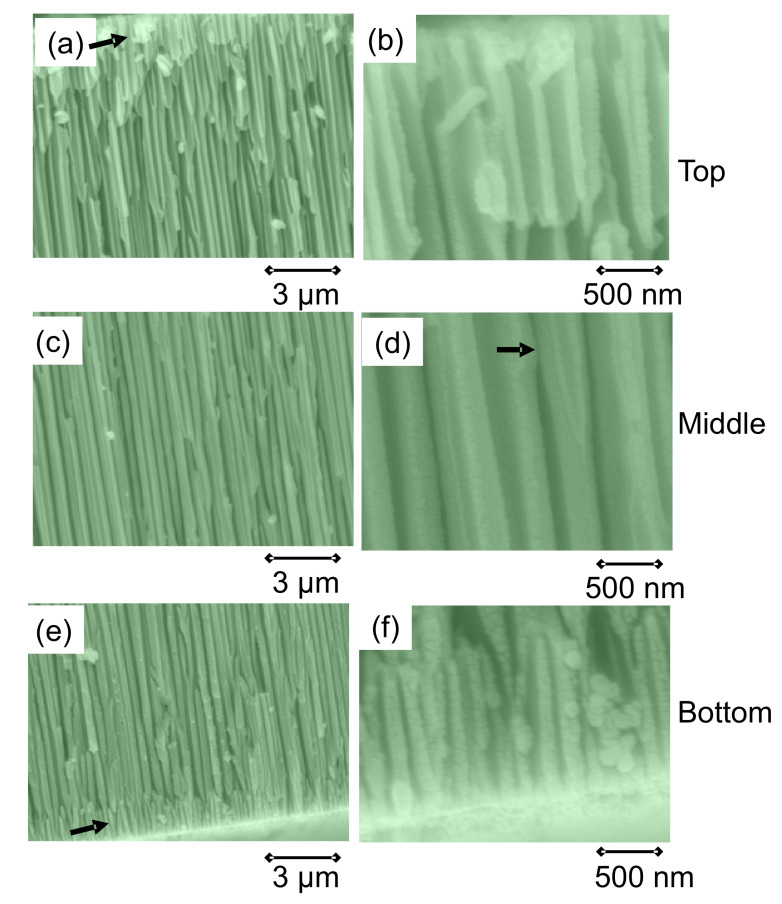
SEM analysis of the cross section of a commercial AAO template with 100 nm nominal pore diameter. The magnification of images (**a**,**c**,**e**) is 10,000×, and images (**b**,**d**,**f**) have a magification of 50,000×. A top layer and a bottom layer (arrows in panels (**a**,**e**)), which is about 1–1.5 μm thick, showing an irregular arrangement of the pores, can be clearly seen. The arrow in panel (**d**) points to a constriction, which is typically found in the middle section of the templates, defining the nominal pore diameter.

However, the microstructural analysis of the HTsc-filled templates showed that there are hardly differences between experiments using the 20 nm or 100 nm templates. This was also seen by other researchers trying to prepare PbTiO${}_{3}$ nanotubes with these AAO templates [80]. A typical result of our analysis is shown in Figure 7. The size of the pores in the commercial templates is always ∼100 nm (the pore diameters on the top surface tend to be somewhat smaller as at the bottom surface, and, in the center section, the pore size is much larger [76]), and only some constrictions in the center of the template define the smaller nominal diameter, as indicated by arrows. Thus, we have to conclude that the pore diameters measured were mostly larger than the nominal values, especially for the templates with a nominal pore diameter of 20 nm. As this is fully reasonable for the intended use of the AAO templates as nanofilters, we must conclude here that nanowires can only be fabricated with a diameter of ∼150 nm using the commercial templates.

**Figure 8:**
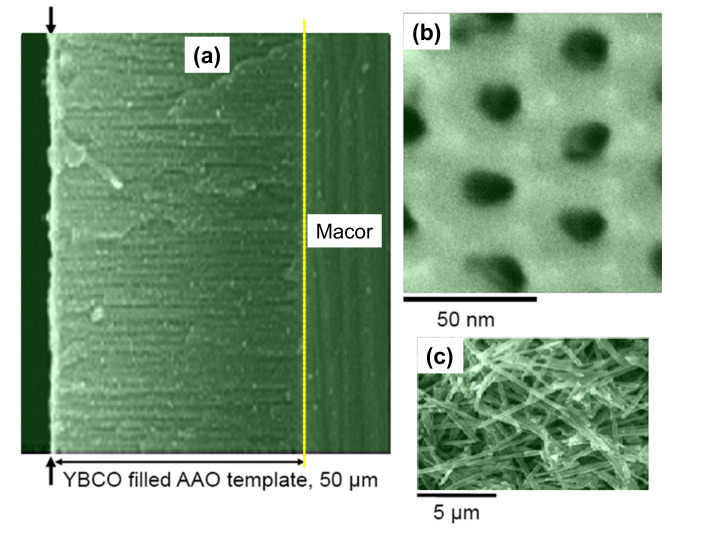
(**a**) SEM cross-sectional view of a YBCO-filled, commercial Whatman anodisc AAO [37] template. For easier handling in the experimetal procedures, the AAO template was glued onto a Macor™ ceramic plate. The arrows mark the still remaining top layer of YBCO material, and the yellow line indicates the template bottom. (**b**) SEM top surface view of the filled template. The YBCO material appears dark; (**c**) shows extracted YBCO nanowires from the templates after NaOH etching. The length of the extracted nanowires is ranging between 2 and 10 μm, but the nanowire surfaces have many structural defects.

Figure 8 shows the microstructural analysis of YBCO-filled AAO templates (a,b) and the extracted nanowires (c). After several unsuccessful experiments, the YBCO nanowires could be separated from the AAO template by chemical etching with a 4 mol/l NaOH solution (holding time for at least 1 h). The diameter of the nanowires is not homogeneous [76], but the pieces of the nanowires obtained are free of cracks. The length of the nanowire pieces is typically about 2–10 μm.

**Figure 9:**
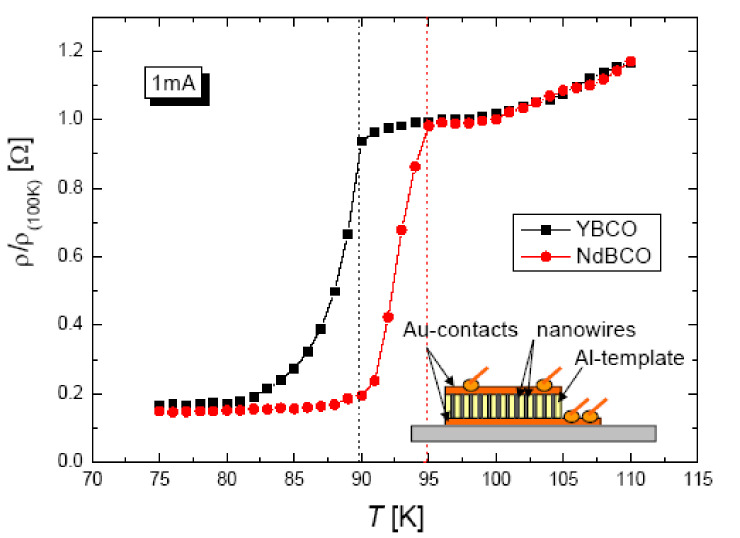
Resistance measurements on YBCO and NdBCO nanowire arrays fabricated using commercial AAO templates. The applied current was 1 mA. NdBCO shows the onset of the superconducting transition at 95 K, while YBCO has a $T_{c}$ of 88 K (indicated by red and black dashed lines). The inset illustrates the arrangement of the samples and the electrical contacts for a four-probe measurement. Au-films were evaporated onto the polished top and bottom surfaces of the AAO template to provide contacts to the nanowire array.

Fianally, Figure 9 presents the electric transport measurements performed on such YBCO and NdBCO nanowire arrays [77]. The measured superconducting transition temperatures, $T_{c}$, are 89 K (YBCO) and 94 K (NdBCO), respectively. So, both values correspond to the $T_{c}$-values of the bulk counterparts, even though the transition widths are relatively broad. The inset in Figure 9 illustrates the electrical connections to the nanowire array provided by two Au layers on top and bottom of the AAO template.

Thus, the melting of HTSc is a feasible method to fill the AAO templates in a straightforward experiment. Any effect of Al diffusion to affect the transition temperature of the resulting HTSc nanowires was not observed, but the removing of the template material to extract the HTSc nanowires is a tedious process, so it is more useful to analyze the entire nanowire arrays in the electric and magnetic measurements.

### 3.4. Templates to Introduce Defect Structures in thin Films

In this section, we discuss the common work of the groups of Piraux and Moshchalkov. Here, the AAO templates were not intended to be filled with superconducting material but served as structured substrates to introduce defects in a superconducting film evaporated on top of the template. This work was initiated by a first work of Welp et al. [81] on Nb films on top of the AAO templates and is similar to previous work covering AAO templates with, e.g., permalloy films to achieve a patterned media for magnetic storage. In case of a superconducting film on top of the AAO membrane, there is a striking similarity of the hexagonal vortex lattice with the hexagonal lattice of the AAO pores. Thus, one can expect matching effects as the distances in the vortex lattice are tunable on applying magnetic field perpendicular to the template surface according to

(1) $$B=2\Phi_{0}/\sqrt{3}a_{0}^{2}\;\;\;,$$

with $\Phi_{0}$ denoting the magnetic flux quantum, *B* the external magnetic field, and $a_{0}$ the intervortex spacing [82].

**Figure 10:**
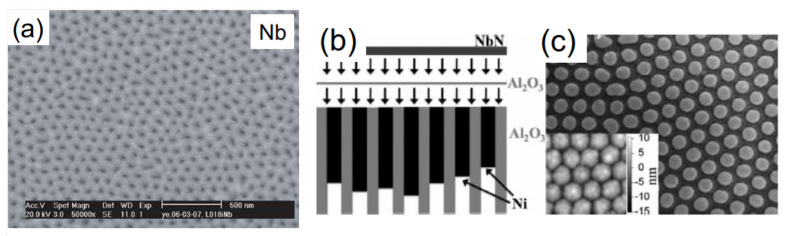
(**a**) Image of a 50-nm thick Nb film evaporated on top of an AAO template. The porous structure is inherited from the AAO template beneath, Reprinted with permission Ref. [46]. Copyright 2006 Springer Nature. (**b**) Schematic representation showing the deposition of a 10 nm thick Al${}_{2}$O${}_{3}$ insulating layer and of a 25 nm thick NbN film on the extremities of an array of Ni nanowires embedded in a nanoporous alumina template; (**c**) presents a SEM image of the surface of the filled template after the polishing step. The inset shows an AFM topography image of the surface after the evaporation of the insulating layer. Reprinted with permission from Ref. [50]. Copyright 2009 Elsevier.

In Refs. [46,47,48,49,50,51] large scale superconducting antidot arrays were grown from Si-supported anodized alumina substrates. Figure 10a presents an SEM micrograph of a Nb film on top of an AAO template, and (b) gives a schematic representation showing the deposition of a 10 nm thick Al${}_{2}$O${}_{3}$ insulating layer and of a 25 nm thick NbN film on the extremities of an array of Ni nanowires embedded in a nanoporous alumina template. Finally, (c) presents a SEM image of the surface of the filled template after the polishing step. The inset shows an AFM topography image of the surface after the evaporation of the insulating layer.

**Figure 11:**
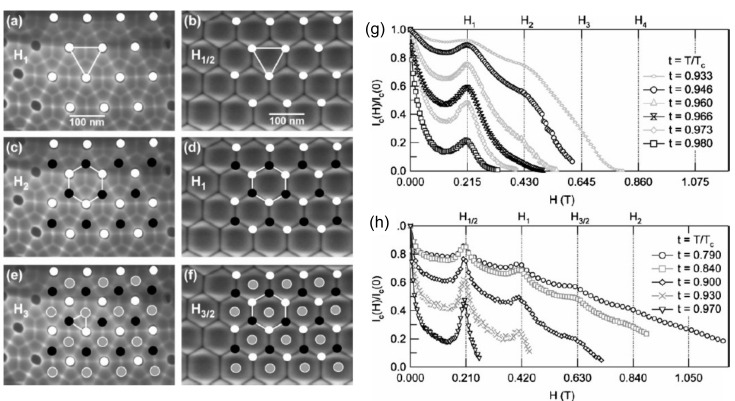
Vortex patterns for a 50-nm thick Nb film with a triangular lattice of antidots of ∼100-nm spacing at (**a**) $H_{1}^{\mathrm{top}}=$ 215 mT, (**c**) $H_{1}^{\mathrm{top}}=$ 430 mT, and (**e**) $H_{1}^{\mathrm{top}}=$ 645 mT, and the 50-nm thick sample evaporated under 30${}^{\circ }$ on the barrier layer surface at (**b**) $H_{1/2}^{\mathrm{bottom}}=$ 210 mT, (**d**) $H_{1/2}^{\mathrm{bottom}}=$ 420 mT, and (**f**) $H_{1/2}^{\mathrm{bottom}}=$ 630 mT. Normalized critical-current curves versus magnetic field for different reduced temperatures of (**g**) a 50-nm Nb film with triangular lattice of antidots (evaporated perpendicularly onto the top surface) and (**h**) a 50-nm Nb film with a quasi-hexagonal thickness modulation (evaporated under 30${}^{\circ }$ from normal onto the bottom surface). Reprinted with permission from Ref. [49]. Copyright 2009 Wiley-VCH.

In Figure 11a–f, the vortex patterns are shown, and, in Figure 11g,h, corresponding magnetic data (normalized critical current versus magnetic field) are presented, taken from Ref. [49]. The fields $H_{1/2}$, $H_{1}$, etc., are the matching fields seen in the magnetic data, and images in Figure 11a–f show the corresponding vortex arrangements. The transport and magnetization measurements performed in these works [46,47,48,49,50,51] have established the existence of pronounced matching effects (peaks in the *M*-*H*-diagrams) in applied magnetic fields up to 700 mT at temperatures as low as 5.7 K. The critical current density of the films was shown to be increased by two orders of magnitude.

A similar experiment was carried out by Ye et al. [83], who fabricated a Co-nanorod array by the AAO template approach and covered this one with a Pb/Bi superconducting film. Hysteretic superconducting properties and increased critical current density in the superconducting film were revealed in the magnetization measurements, and, for explanation of these effects, the domain structure of the Co-nanowire array was employed. This work nicely demonstrates the importance of magnetic pinning centers in order to increase the flux pinning properties of superconducting materials.

To conclude this section, we may state that the use of AAO templates (filled with magnetic material, or in pure form) to fabricate superconducting films on an anti-dot lattice brought up interesting experiments concerning the flux pinning properties of mostly, conventional metallic superconductors. However, the fabrication of so-called hybrid magnetic/superconducting systems [84] would also be possible using HTSc films plus AAO templates filled with magnetic material to study the flux pinning enhancements.

## 4. Discussion

In general, the templating approach to prepare superconducting nanowires has produced several interesting results published in the literature, but mainly on the conventional metallic superconductors, where the AAO material can be removed from the nanowires straightforwardly by etching. In addition, for these materials, the parameters of the pores enable to produce true 1D nanowires, so the influences of the thermal activated phase slips (TAPS) and the quantum phase slips (QPS) could be nicely demonstrated. More recent experiments also addressed the effects of $T_{c}$ enhancement by nanostructuring, and novel filling approaches (metallic-flux nanonucleation [8]) were developed, so we may expect more interesting physics to be revealed in various other, nano-patterned superconducting materials in the near future. The other approach of producing superconducting films on top of the AAO membranes producing antidot lattices to increase flux pinning also generated very nice results showing matching effects between the hexagonal pore lattice and the magnetic field-tunable flux-line lattice.

For the HTSc materials, the problems with separating the nanowires from the templates must be pointed out as the main problem obscuring the wider use of the AAO templates to produce HTSc nanowires. Thus, electrospinning [85,86,87] and solution-blow spinning [88,89] have completely overrun the template approach, enabling the growth of longer and homogeneous HTSc nanowire fabrics, from which individual pieces can easily be cut off using FIB [72,90]. Furthermore, the nanowire fabrics themselves have interesting electric and magnetic properties [91,92,93,94,95,96,97,98,99,100,101], which may lead to specific applications which are not possible with other types of HTSc materials.

**Figure 12:**
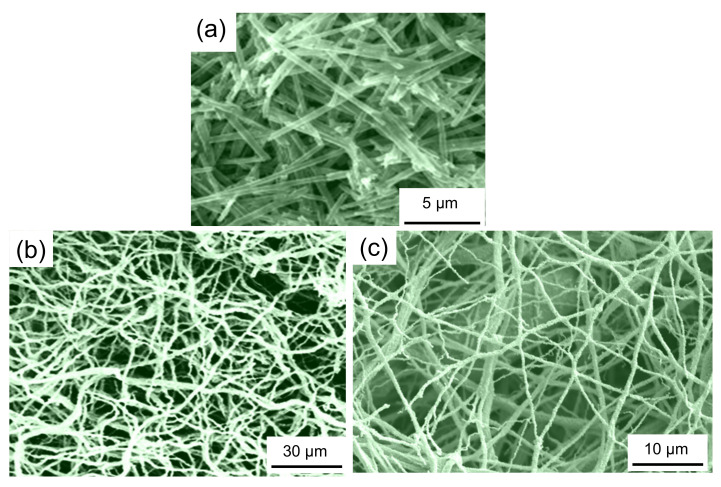
Comparison of HTSc nanowires fabricated using the AAO template approach ((**a**), YBCO) [77] with such prepared by the electrospinning technique ((**b**), Bi-2212) as measured in Ref. [95] and solution blow-spun nanowires ((**c**), YBCO) [99]. Note the obvious difference in the length scale.

Figure 12a–c present a comparison of AAO-templated YBCO nanowires (a, taken from Ref. [77]), electrospun Bi-2212 nanowires (b, data from Saarbrücken) and solution blow-spun YBCO nanowires (c, Reference [99]). Note here the much longer length of the individual nanowires prepared by electrospinning (the resulting nanowires produced by solution blow-spinning are quasi identical) as compared to the ones by AAO templating. Furthermore, the separation of individual nanowire pieces from the prepared nanowire fabric is simple, applying the FIB technique. The problem with the spinning approach remains, however, in the large nanowire diameter of 200–500 nm, which is far too big to obtain true, 1D-HTSc nanowires. Thus, future research is required to find templates suitable to produce HTSc nanowires in the 10 nm range.

Several methods were applied in the literature to analyze the microstructure of the template-prepared nanowires. Among these are SEM, EDX, (high-resolution) TEM, selected area electron diffraction (SAED), and XRD, but a detailed investigation concerning the grain boundary misorientation and an eventual texture is missing in the literature. In case of the conventional superconductors, there was not much work done in this direction, and, for HTSc nanowires, the problems arising due to the difficulties in removing the nanowires from the templates have prevented the application of an analysis technique, such as the electron backscatter diffraction (EBSD). In contrast, on electrospun nanowires, EBSD and its further development, transmission Kikuchi diffraction (TKD, or some times called t-EBSD) [102] has revealed the existence of a fiber-like texture of the superconducting Bi-2212 and ferromagnetic (La,Sr)MnO${}_{3}$ nanowires [103,104]. Another unsolved issue concerning the sol-gel-derived, templated HTSc nanowires is the analysis of the eventually remaining solvent within the template, as no thermogravimetric data are published in the literature. Furthermore, magneto-optic imaging (MO [105,106,107]) of flux penetration into the superconducting nanostructures was not attempted in any experiment, neither magnetic force microscopy (MFM), as was done on magnetic nanowires [108]. In this direction, there is still plenty of work to be done in the future.

For the analysis of the superconducting properties, electric transport, and magnetic measurements on the nanowires are required, preferably on individual nanowires. In the case of HTSc materials, the fabrication of proper electric contacts to an individual nanowire is quite complicated due to the nanowire surfaces, which are inhomogeneous. So, even for the electrospun nanowires, which can be handled by FIB, the evaporation of Pt contacts required several attempts to produce reasonably good electric contacts with low resistance [90]. Another problem prevails with the magnetic data. The usually employed measurement systems (SQUID, VSM, or AC susceptibility) always require a substantial amount of superconducting material, which excludes the measurement of an individual nanowire. Thus, the use of a specifically developed, low-temperature cantilever magnetometer would be highly desirable [109] to be applied in the research on superconducting nanowires.

In the review of Piraux on magnetic nanowires [36], it was demonstrated that the electrodeposition technique, together with the AAO templates, enables the fabrication of a variety of nanowire-based architectures. Furthermore, the template-assisted electrodeposition provides the control of the chemical composition (e.g., multi-layered nanowires), the density/spatial control (arrangements of the nanowires within the template, e.g., crossed nanowires), and the shape control (nanowires, nanotubes, nanowires with constrictions, aspect ratio). Many of these features are welcome for magnetic nanowires; for example, nanowires with selected aspect ratio can be produced for the use as magnetic elements in ferrofluids [110,111]. In addition, the magnetic reversal properties of the nanowires is directly influenced by the shape and aspect ratio [108,112,113,114,115,116]. Furthermore, this excellent control enables tuning of the magnetic, magneto-transport, and thermoelectric properties of the resulting nanowires or nanowire arrays. Most of these advantages have not (yet) been explored for superconducting nanowires.

However, there is still a demand for even smaller nanowire diameters for both conventional metallic, as well as the HTSc superconducting, materials. Thus, a variety of ideas was already discussed in the literature using other template materials, influenced by the progress of nanotechnology. These ideas comprise DNA sections as templates [117], molecular templates [118], and biomimetic templates from chitosan [119]. Other proposals made include self-assembled Si templates [120], combinations of porous Si with high-resolution electron beam lithography [121], block copolymer double gyroid-derived ceramic templates [122], and zeolite [123]. Using these new approaches, new superconducting materials were also obtained in nanowire form, including MgB${}_{2}$ and δ3-MoN [124]. Very recently, DNA origami as template material was used by Shani et al. [125] to prepare NbN nanowires. All these new approaches clearly demonstrate that the templating technique is still very interesting to fabricate superconducting nanowires with smaller and smaller diameters, and more superconducting materials can also be produced in nanowire form, which may lead to more new interesting physics. Other developments concern the growth technique itself, such as vapor-solid growth [126] and the metallic-flux nanonucleation technique [8].

Finally, we give some comments on possible applications of nanowires, nanowire arrays, and nanowire fabrics. As the conventional metallic superconducting nanowires can be easily separated from the templates, these nanowires can be applied as sensor elements or connecting wires in electronic circuits, as mentioned already at the beginning. An application of a superconducting nanowire array still within its template has not yet been described in the literature. For the templated HTSc nanowires, there are no applications envisaged in the literature, nor for the nanowire arrays. Undoubtedly, in contrast, the electrospun or blow-spun HTSc nanowires have a variety of possible applications in the form of the nanowire network fabrics, as described in Refs. [127,128,129], but the superconducting transition temperature, $T_{c}$, being too close to the application temperature of 77 K (temperature of liquid nitrogen), still hinders the planned applications, such as the “superconducting carpet” [127] or applications, as shielding materials until nanowires with higher $T_{c}$ (and, of course, higher critical current density, $j_{c}$) are fabricated in nanowire form.

## 5. Conclusions

To conclude, the preparation of superconducting nanowires using the AAO template route is working successful for conventional metallic nanowires, whereas, for the preparation of HTSc nanowires, the problem of removing the nanowires from the templates prevails. Furthermore, the preparation of superconducting thin films on top of the AAO templates gave interesting results due to matching effects of the pore lattice with the flux line lattice. Again, the same approach does not work properly for the HTSc as Al${}_{2}$O${}_{3}$ is not a suitable substrate for HTSc thin film growth. Thus, the AAO template approach for the fabrication of nanowires of conventional metallic and HTSc superconducting materials is feasible to grow nanowires, but there is still a demand for smaller nanowire diameters, which may reveal new physics, e.g., concerning a possible increase of $T_{c}$ or the stabilization of uncommon crystallographic modifications in nanowire form, so different types of templates are discussed in the literature.

## References

[1] Guo Y., Zhang Y.F., Bao X.J., Han T.Z., Tang Z., Zhang L.X., Zhu W.G., Wang E., Niu Q., Qiu Z.Q. (2004). Superconductivity modulated by quantum size effects. Science.

[2] Zgirski M., Riikonen K.P., Touboltsev V., Arutyunov K. (2005). Size dependent breakdown of superconductivity in ultranarrow nanowires. Nano Lett..

[3] Charnaya E., Tien C., Lin K., Wur C., Kumzerov Y.A. (1998). Superconductivity of gallium in various confined geometries. Phys. Rev. B.

[4] Watson J. (1970). Transition temperature of superconducting indium, thallium, and lead grains. Phys. Rev. B.

[5] Li W.H., Yang C., Tsao F., Wu S., Huang P., Chung M., Yao Y. (2005). Enhancement of superconductivity by the small size effect in in nanoparticles. Phys. Rev. B.

[6] Ohshima K., Fujita T. (1986). Enhanced superconductivity in layers of Ga fine particles. J. Phys. Soc. Jpn..

[7] Hagel J., Kelemen M., Fischer G., Pilawa B., Wosnitza J., Dormann E., v. Löhneysen H., Schnepf A., Schnöckel H., Neisel U. (2002). Superconductivity of a crystalline Ga 84-cluster compound. Low Temp. Phys..

[8] Moura K.O., Pirota K.R., Béron F., Jesus C.B.R., Rosa P.F.S., Tobia D., Pagliuso P.G., de Lima O.F. (2017). Superconducting Properties in Arrays of Nanostructured *β*-Gallium. Sci. Rep..

[9] Narlikar A.V. (2014). Superconductors.

[10] Bennemann K.H., Ketterson J.B. (2008). Superconductivity.

[11] Tinkham M., Lau C.N. (2002). Quantum limit to phase coherence in thin superconducting wires. Appl. Phys. Lett..

[12] Haviland D. (2010). Quantum phase slips. Nat. Phys..

[13] Bezryadin A. (2013). Superconductivity in Nanowires.

[14] Altomare F., Chang A.M. (2013). One-Diemsional Superconductivity in Nanowires.

[15] Awschalom D., Berggren K.K., Bernien H., Bhave S., Carr L.D., Davids P., Economou S.E., Englund D., Faraon A., Fejer M. (2021). Development of Quantum InterConnects for Next-Generation Information Technologies. PRX Quantum.

[16] Natarajan C.M., Tanner M.G., Hadfield R.H. (2012). Superconducting nanowire single-photon detectors: Physics and applications. Supercond. Sci. Technol..

[17] Korzh B.A., Zhao Q.-Y., Frasca S., Allmaras J.P., Autry T.M., Bersin E.A., Colangelo M., Crouch G.M., Dane A.E., Gerrits T. (2020). Demonstrating sub-3 ps temporal resolution in a superconducting nanowire single-photon detector. Nat. Photonics.

[18] You L. (2020). Superconducting nanowire single-photon detectors for quantum information. Nanophotonics.

[19] Steinhauer S., Gyger S., Zwiller V. (2021). Progress on large-scale superconducting nanowire single-photon detectors. Appl. Phys. Lett..

[20] Friedl M., Cerveny K., Weigele P., Tütüncüoglu G., Martí-Sánchez S., Huang C., Patlatiuk T., Potts H., Sun Z.H., Hill M.O. (2018). Template-assisted scalable nanowire networks. Nano Lett..

[21] Saxena A.K. (2012). High-Temperature Superconductors.

[22] Huczko A. (2000). Template-based synthesis of nanomaterials. Appl. Phys. A.

[23] Zhang M., Bando Y., Wada K. (2000). Silicon dioxide nanotubes prepared by anodic alumina as templates. J. Mater. Res..

[24] Yin A.J., Li J., Jian W., Bennett A.J., Xu J.M. (2001). Fabrication of highly ordered metallic nanowire arrays by electrodeposition. Appl. Phys. Lett..

[25] Kline T.R., Tian M., Wang J., Sen A., Chan M.W.H., Mallouk T.E. (2006). Template-grown Metal Nanowires. Inorg. Chem..

[26] Cao G., Liu D. (2008). Template-based synthesis of nanorod, nanowire, and nanotube arrays. Adv. Colloid Interface.

[27] Bae C., Yoo H., Kim S., Lee K., Kim J., Sung M.M., Shin H. (2008). Template directed synthesis of oxide nanotubes: Fabrication, characterization, and applications. Chem. Mater..

[28] Piraux L., Dubois S., Demoustier-Champagne S. (1997). Template synthesis of nanoscale materials using the membrane porosity. Nucl. Instr. Methods Phys. Res. B.

[29] Dubois S., Michel A., Eymery J.P., Duvail J.L., Piraux L. (1999). Fabrication and properties of arrays of superconducting nanowires. J. Mater. Res..

[30] Schwarzacher W., Kasyutich O.I., Evans P.R., Bardyshire M.G., Yi G., Fedosyuk V.M., Rousseaux F., Cambril E., Decanini D. (1999). Metal nanostructures prepared by template electrodeposition. J. Magn. Magn. Mater..

[31] Yi G., Schwarzacher W. (1999). Single crystal superconductor nanowires by electrodeposition. Appl. Phys. Lett..

[32] Shingubara S. (2003). Fabrication of Nanomaterials Using Porous Alumina Templates. J. Nanoparticle Res..

[33] Benfield R.E., Grandjean D., Dore J.C., Esfahaniana H., Wu Z.H., Kröll M., Geerkens M., Schmid G. (2004). Structure of assemblies of metal nanowires in mesoporous alumina membranes studied by EXAFS, XANES, X-ray diffraction and SAXS. Faraday Discuss..

[34] Zhang J., Kielbasa J.E., Carroll D.L. (2010). Controllable fabrication of porous alumina templates for nanostructures synthesis. Mat. Chem. Phys..

[35] Abdul M.M.J., Losic D., Voelcker N.H. (2013). Nanoporous anodic aluminium oxide: Advances in surface engineering and emerging applications. Prog. Mater. Sci..

[36] Piraux L. (2020). Magnetic Nanowires. Appl. Sci..

[37] Anodisc™ Membranes, Frameless. www.whatman.com.

[38] Wang J., Tian M., Kumar N., Mallouk T.E. (2005). Controllable Template Synthesis of Superconducting Zn Nanowires with Different Microstructures by Electrochemical Deposition. Nano Lett..

[39] Xu J., Liu X., Li Y. (2004). Single crystalline YBa_2_Cu_3_O_7-δ_ nanowires from a template-assisted sol-gel route. Mater. Chem. Phys..

[40] Li P.G., Fu X.L., Chen L.M., Zhang H.Y., Li L.H., Tang W.H. (2005). Fabrication and characterization of YBa_2_Cu_3_O_y_ Superconducting Nanowires. Chin. Phys. Lett..

[41] Koblischka M.R., Koblischka-Veneva A., Skumryev V., Muralidhar M. (2012). YBCO and NdBCO nanowires grown by the alumina template method. Superconductivity: Recent Developments and New Production Technologies.

[42] Pang Y.T., Meng G.W., Zhang L.D., Shan W.J., Gao X.Y., Zhao A.W., Mao Y.Q. (2002). Arrays of ordered Pb nanowires with different diameters in different areas embedded in one piece of anodic alumina membrane. J. Phys. Cond. Mater..

[43] Djokic S.S. (2010). Modern Aspects of Electrochemistry 48: Electrodeposition. Theory and Practice.

[44] Machado A.J.S., Moehlecke S., Kopelevich Y., Robin A., dos Santos C.A.M. (2000). Superconducting YBa_2_Cu_3_O_7-δ_ films on SrTiO_3_ by electrodeposition process. Physica C.

[45] Phok S., Spagnol P.D., Chaudhuri T., Bhattacharya R.N. (2005). Superconducting YBCO Films Prepared by Electrodeposition and Spray Pyrolysis. MRS Online Proc. Lib..

[46] Vinckx W., Vanacken J., Moshchalkov V.V., Mátéfi-Tempfli S., Mátéfi-Tempfli M., Michotte S., Piraux L. (2006). Vortex pinning in superconducting Nb thin films deposited on nanoporous alumina templates. Eur. Phys. J. B.

[47] Vinckx W., Vanacken J., Moshchalkov V.V., Mátéfi-Tempfli S., Mátéfi-Tempfli M., Michotte S., Piraux L., Ye X. (2007). High field matching effects in superconducting Nb porous arrays catalyzed from anodic alumina templates. Physica C.

[48] Vanacken J., Vinckx W., Moshchalkov V.V., Mátéfi-Tempfli S., Mátéfi-Tempfli M., Michotte S., Piraux L., Ye X. (2008). Vortex pinning in superconductors laterally modulated by nanoscale self-assembled arrays. Physica C.

[49] Hallet X., Mátéfi-Tempfli M., Michotte S., Piraux L., Vanacken J., Moshchalkov V.V., Mátéfi-Tempfli S. (2009). Quasi-Hexagonal Vortex-Pinning Lattic Using Anondized Aluminium Oxide Nanotemplates. Small.

[50] Hallet X., Mátéfi-Tempfli M., Michotte S., Piraux L., Vanacken J., Moshchalkov V.V., Mátéfi-Tempfli S. (2009). High magnetic field matching effects in NbN films induced by template grown dense ferromagnetic nanowires arrays. Appl. Phys. Lett..

[51] Piraux L., Hallet X. (2012). Artificial vortex pinning arrays in superconducting films deposited on highly ordered anodic alumina templates. Nanotechnology.

[52] Michotte S., Mátéfi-Tempfli M., Piraux L. (2003). 1D-transport properties of single superconducting lead nanowires. Physica C.

[53] Li C., Zheng M., Li M., Zhu C., Li M., Wang X., Li Z., Shen W. (2013). Template-based sputtering method for vertically aligned tin nanotube arrays: From fabrication to superconductivity. Thin Solid Films.

[54] De Menten de Horne F., Piraux L., Michotte S. (2005). Fabrication and physical properties of multilayered superconducting nanowires. Appl. Phys. Lett..

[55] Michotte S., Mátéfi-Tempfli M., Piraux L. (2003). Investigation of superconducting properties of nanowires prepared by template synthesis. Supercond. Sci. Technol..

[56] Piraux L., Encinas A., Vila L., Mátéfi-Tempflii S., Mátéfi-Tempflii M., Darques M., Elhoussine F., Michotte S. (2005). Magnetic and Superconducting Nanowires. J. Nanosci. Nanotechnol..

[57] Fusil S., Piraux L., Mátéfi-Tempfli S., Mátéfi-Tempfli M., Michotte S., Saul C.K., Pereira L.G., Bouzehouane K., Cros V., Deranlot C. (2005). Nanolithography based contacting method for electrical measurements on single template synthesized nanowires. Nanotechnology.

[58] Zhang X.Y., Dai J.Y. (2004). Fabrication and magnetic behaviour of superconductor nanowire arrays. Nanotechnology.

[59] Brongersma S., Pothuizen J., Verweij E., Koeman N., Groot D.G., Griessen R. (1993). Multiple maxima in the field dependent magnetisation of superconducting Nb/Cu multilayers. J. Alloys Compd..

[60] Ziese M., Esquinazi P., Wagner P., Adrian H., Brongersma S.H., Griessen R. (1996). Matching and surface barrier effects of the flux-line lattice in superconducting films and multilayers. Phys. Rev. B.

[61] De Menten de Horne F., Piraux L., Michotte S. (2009). Electroless template grown superconducting lead and tin nanotubes. Nanotechnology.

[62] Wang H., Wang J., Tian M., Bell L., Hutchinson E., Rosario M.M., Liu Y., Amma A., Mallouk T. (2004). Metallic contacts with individual nanowires prepared by electrochemical deposition and the suppression of superconductivity in ultrasmall grains. Appl. Phys. Lett..

[63] De Haas W.J., Voogd J. (1929). On the superconductivity of the gallium. Commun. Phys. Lab. Univ. Leiden.

[64] Roberts B.W. (1976). Survey of superconductive materials and critical evaluation of selected properties. J. Phys. Chem. Ref. Data.

[65] Haruyama J., Tokita A., Kobyashi N., Nomura M., Miyadai S., Takazama K., Takeda A., Kanda Y. (2004). End-bonding multiwalled carbon nanotubes in alumina templates: Superconducting proximity effect. Appl. Phys. Lett..

[66] Sharma D., Kumar R., Awana V.P.S. (2013). DC and AC susceptibility study of sol-gel synthezied Bi_2_Sr_2_CaCu_2_O_8+δ_ superconductor. Ceram. Int..

[67] Deguchi Y., Kikuchi H., Mori N., Yamada Y., Atsumi T., Yoshida K., Ishibashi T. (2013). Fluctuation-conductivity characterization of superconducting Bi_2_Sr_2_CaCu_2_O_8+δ_ thinfilms prepared by the metal-organic decomposition method. Phys. Proc..

[68] Lu X., Wang T., Qi Y. (2016). Crystalline characteristics and superconducting properties of Bi2212 thin films by Pechini sol-gel method: Effect of heating rate on the film growth. J. Sol-Gel Sci. Technol..

[69] Liu X., Zhao G. (2018). One step preparation of photosensitive Bi_2_Sr_2_CaCu_2_O_8+x_ films and their fine patterns by a photosensitive sol-gel method. Supercond. Sci. Technol..

[70] Dadras S., Aawani E. (2015). Fabrication of YBCO nanowires with anodic aluminium oxide (AAO) template. Physica B.

[71] Thomsen C., Cardona M., Gegenheimer B., Liu R., Simon A. (1988). Untwinned single crystals of YBa_2_Cu_3_O_7-δ_: An optical investigation of the a-b anisotropy. Phys. Rev. B.

[72] Reyntjens S., Puers R. (2001). A review of focused-ion beam applications in microsystem technology. J. Micromech. Microeng..

[73] Nishimura Y., Yasuhara Y., Miyashita S., Komatsu H. (1996). In situ observation of crystallization of YBCO via the peritectic reaction. J. Cryst. Growth.

[74] Zhang G., Lu X., Zhang T., Qu J., Wang W., Li X., Yu S. (2006). Microstructure and superconductivity of highly ordered YBa_2_Cu_3_O_7-δ_ nanowire arrays. Nanotechnology.

[75] Lai S.H., Hsu Y.C., Lan M.D. (2008). Synthesis of BSCCO nanowire and its superconductivity. Solid State Commun..

[76] Koblischka M.R., Zeng X.L., Hartmann U. (2016). Commercial alumina templates as base to fabricate 123-type high-*T*_c_ superconducting nanowires. Phys. Stat. Sol. A.

[77] Koblischka M.R., Narlikar A.V. (2017). Growth and characterization of nanowires and –ribbons. Oxford Handbook of Small Superconductors.

[78] Hari Babu N., Reddy E.S., Shi Y., Iida K., Withnell T.D., Cardwell D.A. (2005). Large single grain (RE)-Ba-Cu-O superconductors with nano-phase inclusions. IEEE Trans. Appl. Supercond..

[79] Wolf T., Goldacker W., Obst B., Roth G., Flükiger R. (1989). Growth of thick YBa_2_Cu_3_O_7-x_ single crystals from Al_2_O_3_ crucibles. J. Cryst. Growth.

[80] Rørvik P.M., Tadanaga K., Tatsumisago M., Grande T., Einarsrud M.-A. (2009). Template-assisted synthesis of PbTiO_3_ nanotubes. J. Eur. Ceram. Soc..

[81] Welp U., Xiao Z.L., Jiang J.S., Vlasko-Vlasov V.K., Bader S.D., Crabtree G.W., Liang J., Chik H., Xu J.M. (2002). Superconducting transition and vortex pinning in Nb films patterned with nanoscale hole arrays. Phys. Rev. B.

[82] Brandt E.H. (2002). The vortex lattice in conventional and high-*T*_c_ superconductors. Baraz. J. Phys..

[83] Ye Z., Naugle G.D., Wu W., Lyuksyotov I. (2010). Superconducting properties of Pb/Bi films quench-condensed on a porous alumina substrate filled with Co nanowires. J. Supercond. Nov. Magn..

[84] Baek B., Rippard W.H., Benz S.P., Russek S.E., Dresselhaus P.D. (2014). Hybrid superconducting-magnetic memory device using competing order parameters. Nat. Commun..

[85] Li D., Xia Y.N. (2004). Electrospinning of nanofibers: Reinventing the wheel?. Adv. Mater..

[86] Li D., McCann J.T., Xia Y.N. (2006). Electrospinning: A simple and versatile technique for producing ceramic nanofibers and nanotubes. J. Am. Ceram. Soc..

[87] Wu H., Pan W., Lin D., Li H. (2012). Electrospinning of ceramic nanofibers: Fabrication, assembly and applications. J. Adv. Ceram..

[88] Daristotle J.L., Behrens A.M., Sandler A.D., Kofinas P. (2016). A review of the fundamental principles and applications of solution blow spinning. ACS Appl. Mater. Interfaces.

[89] Cheng B., Tao X., Shi L., Yan G., Zhuang X. (2014). Fabrication of ZrO_2_ ceramic fiber mats by solution blowing process. Ceram. Int..

[90] Karwoth T. (2016). Electronic Transport Measurements on Electrospun High-*T*_c_ Fibers. Master’s Thesis.

[91] Li J.M., Zeng X.L., Mo A.D., Xu Z.A. (2011). Fabrication of cuprate superconducting La_1.85_Sr_0.15_CuO_4_ nanofibers by electrospinning and subsequent calcination in oxygen. CrystEngComm.

[92] Duarte E.A., Quintero P.A., Meisel M.W., Nino J.C. (2013). Electrospinning of superconducting BSCCO nanowires. Physica C.

[93] Duarte E.A., Rudawski N.G., Quintero P.A., Meisel M.W., Nino J.C. (2015). Electrospinning of superconducting YBCO nanowires. Supercond. Sci. Technol..

[94] Zeng X.L., Koblischka M.R., Hartmann U. (2015). Synthesis and characterization of electrospun superconducting (La,Sr)CuO_4_ nanowires and nanoribbons. Mater. Res. Express.

[95] Koblischka M.R., Zeng X.L., Karwoth T., Hauet T., Hartmann U. (2016). Transport and magnetic measurements on Bi-2212 nanowire networks prepared via electrospinning. IEEE Trans. Appl. Supercond..

[96] Koblischka M.R., Zeng X.L., Karwoth T., Hauet T., Hartmann U. (2016). Magnetic properties of electrospun nonwoven superconducting fabrics. AIP Adv..

[97] Zeng X.L., Koblischka M.R., Karwoth T., Hauet T., Hartmann U. (2017). Preparation of granular Bi-2212 nanowires by electrospinning. Supercond. Sci. Technol..

[98] Cena C.R., Torsoni G.B., Zadorosny L., Malmonge L.F., Carvalho C.L., Malmonge J.A. (2017). BSCCO superconductor micro/nanofibers produced by solution blow spinning technique. Ceram Int..

[99] Rotta M., Zadorosny L., Carvalho C.L., Malmonge J.A., Malmonge I.F., Zadorosny R. (2016). YBCO ceramic nanofibers obtained by the new technique of solution blow spinning. Ceram. Int..

[100] Rotta M., Motta M., Pessoa A.L., Carvalho C.L., Ortiz W.A., Zadorosny R. (2019). Solution blow spinning control of morphology and production rate of complex superconducting YBa_2_Cu_3_O_7-x_ nanowires. J. Mat. Sci. Mater. Electron..

[101] Rotta M., Namburi D.K., Shi Y., Pessoa A.L., Carvalho C.L., Durrell J.H., Cardwell D.A., Zadorosny R. (2019). Synthesis of Y_2_BaCuO_5_ nano-whiskers by a solution blow spinning technique and their successful introduction into single-grain, YBCO bulk superconductors. Ceram. Int..

[102] Sneddon G.C., Trimby P.W., Cairney J.M. (2016). Transmission Kikuchi diffraction in a scanning electron microscope: A review. Mater. Sci. Eng. R.

[103] Koblischka-Veneva A., Koblischka M.R., Zeng X.L., Schmauch J., Hartmann U. (2018). TEM and electron-backscatter analysis (EBSD) on superconducting nanowires. J. Phys. Conf. Ser..

[104] Koblischka-Veneva A., Koblischka M.R., Zeng X.L., Schmauch J. (2021). Microstructure analysis of electrospun La_0.8_Sr_0.2_MnO_3_ nanowires using electron microscopy and electron backscatter diffraction. AIP Adv..

[105] Koblischka M.R., Wijngaarden R.J. (1995). Magneto-optical investigations of superconductors. Supercond. Sci. Technol..

[106] Koblischka M.R. (2009). Magnetic Properties of High-Temperature Superconductors.

[107] Jooss C.H., Albrecht J., Kuhn H., Leonhardt S., Kronmüller H. (2002). Magneto-optical studies of current distributions in high-*T*_c_ superconductors. Rep. Prog. Phys..

[108] Sorop T.G., Untiedt C., Luis F., Kröll M., Rasa M., de Jongh L.J. (2003). Magnetization reversal of ferromagnetic nanowires studied by magnetic force microscopy. Phys. Rev. B.

[109] Gross B., Weber D.P., Rüffer D., Buchter A., Heimbach F., Fontcuberta i Morral A., Grundler D., Poggio M. (2016). Dynamic cantilever magnetometry of individual CoFeB nanotubes. Phys. Rev. B.

[110] Monz S., Tschöpe A., Birringer R. (2008). Magnetic properties of isotropic and anisotropic CoFe_2_O_4_-based ferrogels and their application as torsional and rotational actuators. Phys. Rev. E.

[111] Bender P., Günther A., Honecker D., Wiedenmann A., Disch S., Tschöpe A., Michels A., Birringer R. (2015). Excitation of Ni nanorod colloids in oscillating magnetic fields: A new approach for nanosensing investigated by TISANE. Nanoscale.

[112] Granitzer P., Rumpf K., Poelt P., Reichmann A., Hofmayer M., Krenn H. (2007). Magnetization of self-organized Ni-nanowires with peculiar magnetic anisotropy. J. Magn. Magn. Mater..

[113] Granitzer P., Rumpf K., Koshida N., Poelt P., Michor H. (2014). Electrodeposited metal nanotube/nanowire arrays in mesoporous silicon and their morphology dependent magnetic properties. ECS Trans..

[114] Jung J.S., Lim J.H., Choi K.H., Oh S.L., Kim Y.R., Lee S.H., Smith D.A., Stokes K.L., Malkinski L., O’Connor C.J. (2005). CoFe_2_O_4_ nanostructures with high coercivity. J. Appl. Phys..

[115] Byrne F., Prina-Mello A., Whelan A., Mohamed B.M., Davies A., Gun’ko Y.A., Coey J.M.D. (2009). High content analysis of the biocompatibility of nickel nanowires. J. Magn. Magn. Mater..

[116] Pitzschel K., Bachmann J., Martens S., Montero-Moreno J.M., Kimling J., Meier G., Eschrig J., Nielsch K., Görlitz D. (2011). Magnetic reversal of cylindrical nickel nanowires with modulated diameters. J. Appl. Phys..

[117] Hopkins D.S., Pekker D., Goldbart P.M., Bezryadin A. (2005). Quantum Interference Device Made by DNA Templating of Superconducting Nanowires. Science.

[118] Bezryadin A., Goldbart P.M. (2010). Superconducting nanowires fabricated using molecular templates. Adv. Mater..

[119] Hall S.R. (2006). Biomimetic synthesis of high-*T*_c_, type-II superconductor nanowires. Adv. Mater..

[120] Cirillo C., Trezza M., Chiarella F., Vecchione A., Bondarenko V.P., Prischepa S.L., Attanasio C. (2012). Quantum phase slips in superconducting Nb nanowire networks deposited on self-assembled Si templates. Appl. Phys. Lett..

[121] Salvato M., Baghdadi R., Cirillo C., Prischepa S.L., Dolgiy A.L., Bondarenko V.P., Lombardi F., Attanasio C. (2017). NbN superconducting nanonetwork fabricated using porous silicon templates and high-resolution electron beam lithography. Nanotechnology.

[122] Thedford R.P., Beaucage P.A., Susca E.M., Chao C.A., Nowack K.C., van Dover R.B., Gruner S.M., Wiesner U. (2021). Superconducting Quantum Metamaterials from High Pressure Melt Infiltration into Block Copolymer Double Gyroid Dervied Ceramic Templates. Adv. Funct. Mater..

[123] Zhang B., Lyu J., Rajan A., Li X., Zhang X., Zhang T., Dong Z., Pan J., Liu Y., Zhang J. (2018). Giant enhancement of superconductivity in arrays of ultrathin gallium and zinc sub-nanowires embedded in zeolite. Mater. Today Phys..

[124] Buh J., Kovič A., Mrzel A., Jagličić Z., Jesih A., Mihailovic D. (2014). Template synthesis of single-phase *δ*3-MoN superconducting nanowires. Nanotechnology.

[125] Shani L., Tinnefeld P., Fleger Y., Sharoni A., Shapiro B.Y., Shaulov A., Gang O., Yeshurun Y. (2021). DNA origami based superconducting nanowires. AIP Adv..

[126] Hsu Y.J., Lu S.-Y. (2005). Vapor-solid growth of Sn nanowires: Growth mechanism and Superconductivity. J. Phys. Chem. B.

[127] Koblischka M.R., Koblischka-Veneva A. (2018). Porous high-*T*_c_ superconductors and their applications. AIMS Mater. Sci..

[128] Koblischka M.R., Koblischka-Veneva A. (2020). The possible applications of superconducting nanowire networks. Mater. Today Proc..

[129] Gokhfeld D., Koblischka M.R., Koblischka-Veneva A. (2020). Highly porous superconductors: Synethesis, research and prospects. Phys. Metals Metallogr..

